# Variation in breast cancer risk associated with factors related to pregnancies according to truncating mutation location, in the French National BRCA1 and BRCA2 mutations carrier cohort (GENEPSO)

**DOI:** 10.1186/bcr3218

**Published:** 2012-07-03

**Authors:** Julie Lecarpentier, Catherine Noguès, Emmanuelle Mouret-Fourme, Marion Gauthier-Villars, Christine Lasset, Jean-Pierre Fricker, Olivier Caron, Dominique Stoppa-Lyonnet, Pascaline Berthet, Laurence Faivre, Valérie Bonadona, Bruno Buecher, Isabelle Coupier, Laurence Gladieff, Paul Gesta, François Eisinger, Marc Frénay, Elisabeth Luporsi, Alain Lortholary, Chrystelle Colas, Catherine Dugast, Michel Longy, Pascal Pujol, Julie Tinat, GENEPSO, Rosette Lidereau, Nadine Andrieu

**Affiliations:** 1 Biostatistics, Institut Curie, rue d'Ulm 26, Paris cedex 05, 75248, France; 2 Biostatistics, Inserm U900, rue d'Ulm 26, Paris cedex 05, 75248, France; 3 Biostatistics, Mines ParisTech, rue St Honoré 35, Fontainebleau Cedex, 77305, France; 4 Public Health, Institut Curie Hôpital René Huguenin, rue Dailly 35, Saint Cloud, 92210, France; 5 Genetic oncology service, Institut Curie, rue d'Ulm 26, Paris cedex 05, 75248, France; 6 Université Claude Bernard Lyon 1, boulevard du 11 Novembre 1918 43, Villeurbanne cedex, 69622, France; 7 Epidemiological and Public Health, CNRS UMR 5558, rue Raphael Dubois 16, Villeurbanne cedex, 69622, France; 8 Unit of genetic epidemiology and prevention, Centre Léon Bérard, rue Laennec 28, Lyon cedex 08, 69373, France; 9 Unit of oncology, Centre Paul Strauss, rue de la porte de l'Hôpital 3, Strasbourg, 67000, France; 10 Unit of oncology, Institut de Cancérologie Gustave Roussy, rue Édouard Vaillant 114, Villejuif Cedex, 94805, France; 11 Unit Genetics, Inserm U830, rue d'Ulm 26, Paris cedex 05, 75248, France; 12 Université Paris-Descartes, rue de l'Ecole de Médecine 15, Paris, 75006, France; 13 Unit of gynecological pathology, Centre François Baclesse, Avenue Général Harris 3, Caen, 14000, France; 14 Oncogenetics, Centre Georges François Leclerc, rue Professeur Marion 1, Dijon, 21000, France; 15 Medical genetics, Hôpital d'enfants, boulevard Maréchal de Lattre de Tassigny 10, Dijon Cedex, 21034, France; 16 Unit medical genetics and oncology, Hôpital Arnaud de Villeneuve CHU Montpellier, avenue du Doyen Gaston Giraud 371, Montpellier Cedex 5, 34295, France; 17 Unit of oncology, Centre Val d'Aurelle, Avenue des Apothicaires-Parc Euromédecine 208, Montpellier Cedex 5, 34298, France; 18 Unit of medical oncology, Institut Claudius Regaud, rue Pont St Pierre 20, Toulouse, 31300, France; 19 Oncology center for the regional cancer genetics consultation Poitou-Charentes, CH Georges Renon, avenue Charles de Gaulle 40, Niort Cedex, 79021, France; 20 Department of anticipation and monitoring of cancer, Institut Paoli-Calmettes, boulevard Sainte Marguerite 232, BP156, Marseille Cedex 09, 13273, France; 21 Unit of medical genetics and oncology, Inserm UMR 912, boulevard Sainte Marguerite 232, BP156, Marseille Cedex 09, 13273, France; 22 Unit of oncology, Centre Antoine Lacassagne, Avenue Valombrose 33, Nice Cedex 02, 06189, France; 23 Unit of medical oncology, Centre Alexis Vautrin, Avenue de Bourgogne 6, Vandœuvre-lès-Nancy, 54511, France; 24 Unit of gynecologic oncology, Centre Catherine de Sienne, Rue Éric Tabarly 2, Nantes, 44202, France; 25 Unit of genetics oncology, Groupe hospitalier Pitié Salpétrière, boulevard de l'Hôpital 83, Paris Cedex 13, 75651, France; 26 Unit Genetics, Centre Eugène Marquis, avenue Bataille Flandres Dunkerque, Rennes Cedex, 35042, France; 27 Laboratory of molecular genetics, Institut Bergonié, Cours Argonne 229, Bordeaux, 33000, France; 28 Unit of genetics, Hôpital Universitaire, Rue Germont 1, Rouen, 76000, France; 29 Laboratory of genetics, Institut Curie Hôpital René Huguenin, rue Dailly 35, Saint Cloud, 92210, France

## Abstract

**Introduction:**

Mutations in *BRCA1 *and *BRCA2 *confer a high risk of breast cancer (BC), but the magnitude of this risk seems to vary according to the study and various factors. Although controversial, there are data to support the hypothesis of allelic risk heterogeneity.

**Methods:**

We assessed variation in BC risk according to factors related to pregnancies by location of mutation in the homogeneous risk region of *BRCA1 *and *BRCA2 *in 990 women in the French study GENEPSO by using a weighted Cox regression model.

**Results:**

Our results confirm the existence of the protective effect of an increasing number of full-term pregnancies (FTPs) toward BC among *BRCA1 *and *BRCA2 *mutation carriers (≥3 versus 0 FTPs: hazard ratio (HR) = 0.51, 95% confidence interval (CI) = 0.33 to 0.81). Additionally, the HR shows an association between incomplete pregnancies and a higher BC risk, which reached 2.39 (95% CI = 1.28 to 4.45) among women who had at least three incomplete pregnancies when compared with women with zero incomplete pregnancies. This increased risk appeared to be restricted to incomplete pregnancies occurring before the first FTP (HR = 1.77, 95% CI = 1.19 to 2.63). We defined the TMAP score (defined as the Time of Breast Mitotic Activity during Pregnancies) to take into account simultaneously the opposite effect of full-term and interrupted pregnancies. Compared with women with a TMAP score of less than 0.35, an increasing TMAP score was associated with a statistically significant increase in the risk of BC (*P *trend = 0.02) which reached 1.97 (95% CI = 1.19 to 3.29) for a TMAP score >0.5 (versus TMAP ≤0.35). All these results appeared to be similar in *BRCA1 *and *BRCA2*. Nevertheless, our results suggest a variation in BC risk associated with parity according to the location of the mutation in *BRCA1*. Indeed, parity seems to be associated with a significantly decreased risk of BC only among women with a mutation in the central region of *BRCA1 *(low-risk region) (≥1 versus 0 FTP: HR = 0.27, 95% CI = 0.13 to 0.55) (*P*_interaction _<10^-3^).

**Conclusions:**

Our findings show that, taking into account environmental and lifestyle modifiers, mutation position might be important for the clinical management of *BRCA1 *and *BRCA2 *mutation carriers and could also be helpful in understanding how *BRCA1 *and *BRCA2 *genes are involved in BC.

## Introduction

Carriers of mutations in the *BRCA1 *and *BRCA2 *genes are at very high risk of developing breast cancer (BC) and ovarian cancer. Estimates of the lifetime risk of developing BC for *BRCA1 *and *BRCA2 *mutation carriers range from 30% to 80% and from 9% to 84%, respectively [1]. Incomplete penetrance and the range of these risk estimates suggest the existence within families of genetic or shared environmental or lifestyle factors that modify the risk of BC.

Many studies have established that women who had their first full-term pregnancy (FTP) at a young age have a lower risk of BC than nulliparous women or women who had their first FTP when they were older than 30 years of age; additional pregnancies are associated with even lower risks (for example, [2,3]). Long-term breastfeeding is also associated with a decreased risk of BC in the general population [4]. Controversial conclusions have been drawn from studies that have examined the risk of BC associated with incomplete pregnancies. While some older studies found a possible positive association between interrupted pregnancies and BC risk [5-9], the most recent meta-analyses concluded that an increased number of either spontaneous or induced abortions was not associated with an increased BC risk [10-12].

The few studies that have assessed the risk of BC associated with incomplete pregnancies [13-15], breast-feeding [13,16-19] and parity [13,15,16,20-23] among *BRCA1/2 *mutations carriers, have shown inconsistent results. For parity, studies have found either no association [16,20,21] or a positive [15] or negative association [13,22] with BC risk. Among studies which have performed analyses according to the gene mutated, one has reported a differential effect of parity on BC risk [23] and one, a differential effect of age at first FTP [13].

Some authors have suggested that the effect of pregnancies in BC development is related to the breast mitotic activity, driven by estrogen and progesterone exposure [24]. This activity appears high during the first three months of pregnancy and is followed by a dramatic decrease and by the differentiation of breast tissue during the last six months [25]. Although lasting and high mitotic activity and incomplete differentiation of breast tissue may have a critical effect on cells with inherited mutations, no study has assessed the effect of breast mitotic activity during pregnancy in *BRCA1 *and *BRCA2 *mutation carriers.

Genotype-phenotype correlations have been found in both *BRCA1 *and *BRCA2 *showing heterogeneity in BC risk according to the location of the mutation (for example, [26-29]). Moreover, inconsistencies in the effect of pregnancy-related factors among *BRCA1 *and *BRCA2 *mutation carriers between studies could be explained by an additional heterogeneity due to a differential effect of these factors according to location of the mutation. Thus, we first studied the effect of pregnancy-related factors on the risk of BC for *BRCA1 *and *BRCA2 *mutation carriers taken together, and by gene. Then we studied the effect of parity, incomplete pregnancies and breast-feeding for homogeneous regions previously described in our data [30] where a central low BC risk region in *BRCA1 *and *BRCA2 *was confirmed [27,28,31-34], and a new high-risk region in *BRCA2 *was described [30].

## Materials and methods

### Data

The GENEPSO study was initiated in 2000 to estimate the risk of breast, ovarian, and other cancers in *BRCA1 *and *BRCA2 *mutation carriers and to assess potential risk-modifying factors, either lifestyle or genetic. Subjects were ascertained from the family cancer clinics of the Genetic and Cancer Group of Unicancer. Any woman who was known to carry a deleterious mutation in the *BRCA1 *or *BRCA2 *gene was eligible, including those diagnosed with cancer and those currently unaffected. They had to be at least 18 years old, mentally capable of giving informed consent to participate in the study, and had been counseled about their mutation status. The research protocol was approved by the relevant ethics committees, and all participants provided written informed consent.

The study population was based on the women enrolled in the GENEPSO study from 2000 to 2010. A total of 1,337 women (from 987 different families) were recruited, 863 (65%) were *BRCA1 *mutation carriers and 474 (35%) were *BRCA2 *mutation carriers. To assess variation in BC risk according to mutation position, a sample with one subject per family was randomly selected to avoid overmatching on the mutation, except for one family where two related women carried two different mutations and thus were considered independent. Additionally, two women were counted twice because they carried two mutations in *BRCA1 *and *BRCA2*. Thus, 990 women were considered for assessing risk factor main effects and for the analyses by mutation location.

A standardized questionnaire on reproductive factors and lifestyle factors was administered to the study subjects by mail. The questionnaire collected detailed information on pregnancy history. Subjects who indicated that they had at least one pregnancy were asked to provide, for each pregnancy, the month and year when the pregnancy started or was terminated, its duration, and its outcome (live birth, still birth, miscarriage, induced abortion), and the duration of breast-feeding, if applicable.

### Genotyping

The mutation screening strategy was similar for all the clinics, that is, the youngest living affected family member was tested first and, if a *BRCA1 *or/and *BRCA2 *mutation was found, affected and unaffected family members were offered testing. Mutations were defined as deleterious when their putative protein products were truncated, that is, nonsense mutations and frameshift mutations (nucleotide insertions or deletions, large gene rearrangements, and splicing defects). Some mutations, without disruption of the reading frame, were considered deleterious when they were classified deleterious by the ENIGMA group (Evidence-based network for the interpretation of germline mutant alleles)[35].

The full coding sequences and the exon-intron junctions of the *BRCA1 *and *BRCA2 *genes were screened for variants, based on pre-screening (denaturing gradient gel electrophoresis (DGGE), single strand conformation polymorphism (SSCP), protein truncation assay (PTA), denaturing high performance liquid chromatography (dHPLC), high resolution melting (HRM), or enhanced mismatch mutation analysis (EMMA)) and sequencing. Several large rearrangements were identified by large cDNA sequencing, multiplex ligation-dependent probe amplification (MLPA) [36], quantitative multiplex PCR of short fragments (QMPSF) [37], quantitative PCR (qPCR) [38], qPCR HRM [39], EMMA [40], bar code screening [41] or dedicated array comparative genomic hybridization (CGH) [42]. Mutation description was provided by each French laboratory, coded and standardized according to the international nomenclature [See Additional file 1 for the distribution of mutations in the study].

### Statistical methods

The data presented here were analyzed using a modified Cox proportional hazards regression model. Standard Cox regression may lead to biased estimates of the hazard ratio (HR) because the women in this study were taken from high-risk families qualifying for genetic testing. The disease status may, therefore, have affected the likelihood of ascertainment and selection leading to an over-sampling of affected women. To correct for this potential bias, the Cox regression analyses were performed using the weighted regression approach described by Antoniou *et al. *[43]. Individuals were weighted such that the observed BC incidence rates in the study sample were consistent with established BC risk estimates for *BRCA1 *and *BRCA2 *carriers [1]. The affected mutation carriers were underweighted (weights <1) and the unaffected mutation carriers were overweighted (weights >1). The weights were applied to all person-years of each subject in the modified Cox model.

Subjects were followed up from birth and censored at the date of diagnosis, for women who were affected by any cancer, or the date of prophylactic bilateral mastectomy or interview, for unaffected women.

Parity, breast-feeding, incomplete pregnancies, menopausal status and oral contraceptive use changed over time, so it was analyzed as a time-dependent covariate and cumulative over life time. All analyses were stratified by period of birth (before 1940, 1940 to 1949, 1950 to1959, 1960 or later). In addition, because menopausal status, oral contraceptive use and gene may substantially modify the risk of BC and thus be a potential confounder, analyses were adjusted for these factors.

To avoid the potential bias due to BC detected during a pregnancy which may cause a bias either toward or away from the null depending on the effect of pregnancy on the risk of BC, pregnancies were included only if they occurred at least one year before the age at censure. Thus, we excluded ten pregnancies, seven among affected women and three among unaffected women.

To assess the variation of BC risk associated with pregnancies and breast-feeding by location of truncating mutations in *BRCA1 *and *BRCA2*, we used regions previously defined as homogeneous in BC risk by Lecarpentier *et al. *We considered two groups of mutation in *BRCA1*, those located in LR1 (for 'low-risk region in *BRCA1'*: codons 374 to 1161) and those located outside LR1. In *BRCA2*, we considered three groups of mutation in *BRCA2*, those located in LR2 (for 'low-risk region in *BRCA2*': codons 957 to 1827), located in HR2 (for 'high-risk region in *BRCA2*': codons 2546 to 2968) and those located outside LR2 and HR2 [30]. Heterogeneity in risk by mutation location was assessed by testing the interaction between mutation location and the risk factor of interest.

All statistical analyses were two-sided and were performed using the STATA statistical package (version 10; Stata Corporation, College Station TX).

## Results

Characteristics of the whole cohort and of one-woman-per-family cohorts are listed in Table 1. A total of 563 women had been diagnosed with BC at the time of their interview, but only 499 of them were considered as affected in this analysis after censoring. The remaining 838 women were censored at age of diagnosis of ovarian cancer (N = 89), at diagnosis of another cancer (N = 16), at prophylactic bilateral mastectomy (N = 11), or at interview (N = 722). The average age at censoring for the 838 participants without BC was 40.0 years (standard deviation (SD) = 0.4), which is similar to the age at diagnosis of the women with BC (41.0 years, SD = 0.4), although the age at interview was substantially higher for the BC patients, reflecting the pattern of genetic testing among participants. Sampling of one woman per family did not change any characteristic distribution or the average of age at censure (39.8 years, SD = 0.5 and 40.4 years, SD = 0.5, respectively, for women without and with BC). Year of birth, number of full-term and incomplete pregnancies, breast-feeding, menopausal status, and oral contraceptive use are also described. There was a total of 39,666 person-years of observation.

**Table 1:** Characteristics of the cohort study of *BRCA1/2 *mutation carriers.

**Characteristics**		**Whole cohort **						**One woman per family sample cohort**					
			
		**All women** **(N = 1337)**		**With BC** **(N = 499)**		**Without BC (N = 838)**		**All women** **(N = 990)**		**With BC** **(N = 379)**		**Without BC (N = 611)**	
			
		**No**	**%**	**No**	**%**	**No**	**%**	**No**	**%**	**No**	**%**	**No**	**%**
	
Mutation													
	*BRCA1*	863	64.5	332	66.5	531	63.4	635	64.1	240	63.3	395	64.6
	*BRCA2*	474	35.5	167	33.5	307	36.6	355	35.9	139	36.7	216	35.4
Age at interview, years													
	Mean	44.1		49.4		41.0		43.7		48.6		40.7	
	SD	0.3		0.5		0.4		0.4		0.5		0.5	
Age at diagnosis/censoring, years													
	Mean	40.4		41.0		40.0		40.1		40.4		39.8	
	SD	0.3		0.4		0.4		0.3		0.5		0.5	
	<30	196	14.7	34	6.8	162	19.3	142	14.3	29	7.7	113	18.5
	30 to 39	487	36.4	205	41.1	282	33.7	371	37.5	159	42.0	212	34.7
	40 to 49	403	30.1	176	35.3	227	27.1	306	30.9	133	35.1	173	28.3
	50 to 59	180	13.5	67	13.4	113	13.5	126	12.7	47	12.4	79	12.9
	≥60	71	5.3	17	3.4	54	6.4	45	4.5	11	2.9	34	5.6
Year of birth													
	<1950	354	26.5	201	40.3	153	18.3	237	23.9	139	36.7	98	16.0
	1950 to 1959	324	24.2	165	33.1	159	19.0	248	25.1	128	33.8	120	19.6
	1960 to1969	351	26.3	119	23.8	232	27.7	282	28.5	99	26.1	183	30.0
	≥1970	308	23.0	14	2.8	294	35.1	223	22.5	13	3.4	210	34.4
Oral contraceptive use													
	Never	261	19.5	122	24.4	139	16.6	180	18.2	86	22.7	94	15.4
	Ever	1,058	79.1	373	74.7	685	81.7	798	80.6	290	76.5	508	83.1
	Missing	18	1.3	4	0.8	14	1.7	12	1.2	3	0.8	9	1.5
Number of full-term pregnancies													
	0	293	21.9	68	13.6	225	26.8	217	21.9	58	15.3	159	26.0
	1	250	18.7	108	21.6	142	16.9	196	19.8	90	23.7	106	17.3
	2	452	33.8	182	36.5	270	32.2	346	34.9	139	36.7	207	33.9
	≥3	342	25.6	141	28.3	201	24.0	231	23.3	92	24.3	139	22.7
	Missing	0	0.0	0	0.0	0	0.0	0	0.0	0	0.0	0	0.0
Induced abortion													
	0	1,060	79.3	383	76.8	677	80.8	776	78.4	286	75.5	490	80.2
	1	213	15.9	81	16.2	132	15.8	168	17.0	67	17.7	101	16.5
	2	44	3.3	22	4.4	22	2.6	31	3.1	15	4.0	16	2.6
	≥3	12	0.9	8	1.6	4	0.5	9	0.9	7	1.8	2	0.3
	Missing	8	0.6	5	1.0	3	0.4	6	0.6	4	1.1	2	0.3
Spontaneous abortion													
	0	1,085	81.2	387	77.6	698	83.3	791	79.9	293	77.3	498	81.5
	1	173	12.9	78	15.6	95	11.3	144	14.5	64	16.9	80	13.1
	2	50	3.7	22	4.4	28	3.3	34	3.4	12	3.2	22	3.6
	≥3	21	1.6	8	1.6	13	1.6	16	1.6	7	1.8	9	1.5
	Missing	8	0.6	4	0.8	4	0.5	5	0.5	3	0.8	2	0.3
Breast-feeding													
	Never	439	32.8	187	37.5	252	30.1	308	31.1	130	34.3	178	29.1
	Ever	568	42.5	230	46.1	338	40.3	442	44.6	182	48.0	260	42.6
	Missing	37	2.8	14	2.8	23	2.7	23	2.3	9	2.4	14	2.3
	Nulliparous	293	21.9	68	13.6	225	26.8	217	21.9	58	15.3	159	26.0
Menopausal status													
	Premenopausal	1,068	79.9	404	81.0	664	79.2	795	80.3	312	82.3	483	79.1
	Postmenopausal	240	18.0	86	17.2	154	18.4	171	17.3	60	15.8	111	18.2
	Unknown	29	2.2	9	1.8	20	2.4	24	2.4	7	1.8	17	2.8

BC, breast cancer; N, number; SD, standard deviation.

The estimated risks of BC associated with parity, age at first FTP, and history of breast-feeding from the weighted Cox regression analysis are summarized in Table 2, both for the entire sample and for *BRCA1 *and *BRCA2 *mutation carriers separately. We also analyzed the parity according to attained age (40 years or younger versus older than 40 years).

**Table 2:** Risk of breast cancer associated with full-term pregnancies and breast feeding.

Reproductive Factors		One woman per family cohort: 39,666 person-years of follow-up					*BRCA1 *mutation carriers: 25,045 person-years of follow-up					*BRCA2 *mutation carriers: 14,621 person-years of follow-up				
		
		Person-years^a^	No. of cases^a^	HR	95% CI	*P *value	Person-years^a^	No. of cases^a^	HR	95% CI	*P *value	Person-years^a^	No. of cases^a^	HR	95% CI	*P *value
Parity^b^																
	Nulliparous	25,333	58	1.00			16,215	38	1.00			9,118	20	1.00		
	Parous	14,333	321	0.77	0.53-1.13		8,830	202	0.76	0.49-1.19		5,503	119	0.78	0.39-1.55	
No. of full-term pregnancies^b^																
	0	25,333	58	1.00			16,215	38	1.00			9,118	20	1.00		
	1	4,435	90	1.10	0.72-1.68		2,777	58	1.04	0.63-1.72		1,658	32	1.24	0.59-2.61	
	2	5,640	138	0.79	0.52-1.20		3,540	87	0.81	0.50-1.30		2,100	51	0.73	0.35-1.55	
	≥3	4,243	92	0.51	0.33-0.81	<10^-3^	2,498	56	0.52	0.31-0.89	0.02	1,745	36	0.49	0.22-1.10	0.08
	Trend			0.77	0.68-0.88	<10^-3^			0.78	0.67-0.91	<10^-3^			0.74	0.58-0.95	0.02
No. of full-term pregnancies by attained age^b^																
	0	25,333	58	1.00			16,215	38	1.00			9,118	20	1.00		
	1-2 before age 40	8,146	131	1.05	0.68-1.63		5,169	88	1.04	0.62-1.77		2,977	43	1.12	0.53-2.37	
	≥3 before age 40	2,303	40	0.82	0.48-1.41		1,414	29	0.88	0.46-1.66		889	11	0.64	0.23-1.75	
	1-2 after age 40	1,929	97	0.70	0.36-1.36		1,148	57	0.70	0.32-1.50		781	40	0.61	0.19-1.99	
	≥3 after age 40	1,940	52	0.35	0.17-0.70	<10^-3^	1,084	27	0.34	0.15-0.76	0.01	856	25	0.36	0.11-1.16	0.09
Age at first full-term pregnancy^c^																
	< 20 years	1,866	39	1.00			1,217	27	1.00			649	12	1.00		
	20-24 years	7,158	152	0.91	0.55-1.50		4,296	93	0.91	0.52-1.61		2,862	59	0.87	0.32-2.34	
	25-29 years	3,994	85	0.62	0.36-1.06	0.08	2,492	53	0.57	0.31-1.06	0.08	1,502	32	0.63	0.22-1.80	
	≥30 years	1,315	45	0.67	0.36-1.23		825	29	0.64	0.32-1.31		490	16	0.65	0.20-2.13	
	Nulliparous	25,333	58	0.41	0.20-0.85	0.02	16,215	38	0.43	0.19-0.97	0.04	9,118	20	0.35	0.09-1.40	
Breast-feeding^c^																
	Never	5,962	131	1.00			3,644	87	1.00			2,318	44	1.00		
	Ever	7,875	182	1.02	0.76-1.36		4,840	111	0.93	0.66-1.30		3,035	71	1.45	0.84-2.51	
	Nulliparous	25,333	58	0.61	0.37-1.02	0.06	16,215	38	0.62	0.33-1.14		9,118	20	0.53	0.21-1.30	

^a ^Not including missing data. ^b ^Adjusted for menopausal status (yes, no), oral contraceptives (never, ever), and gene mutated (*BRCA1*, *BRCA2*). ^c ^Adjusted for parity (0, 1, 2, ≥3), menopausal status (yes, no), oral contraceptives (never, ever), and gene mutated (*BRCA1*, *BRCA2*). HR hazard ratio; CI confidence interval, No., number.

Overall, compared with nulliparous women, parous women had a slightly lower but non significant risk of BC (HR = 0.77, 95% CI = 0.53 to 1.13). As the number of FTPs increased there was a statistically significant decrease in the risk of BC (*P *trend < 10^-3^). The reduction in risk was estimated with an HR = 0.51 (95% CI = 0.33 to 0.81) for women with at least three FTPs. This association remained significant only for the women who were older than 40 years (for women with at least three FTPs, HR = 0.35, 95% CI = 0.17 to 0.70). Among parous women, age at first FTP seems to be associated with BC risk. Indeed, women who had their first FTP when they were 25 years or older had a lower HR point estimate of BC than women who had their first FTP when they were younger than 20 years (between age 25 and 30 versus before age 20, HR = 0.62, 95% CI = 0.36 to 1.06 and after age 30 versus before age 20, HR = 0.67, 95% CI = 0.36 to 1.23). The reduction in risk associated with parity and age at first FTP was similar for carriers of *BRCA1 *and *BRCA2 *mutations. After adjusting for parity, we observed no association between ever having breast-fed and BC risk, either for the entire sample or separately for *BRCA1 *or *BRCA2 *mutation carriers. There was also no statistically significant association between duration of breast-feeding and BC risk even for long duration (that is, ≥10 months) (data not shown).

The estimated risks of BC associated with incomplete pregnancies, from the weighted Cox regression analysis, are summarized in Table 3. First, HR point estimates suggest an association between incomplete pregnancy (induced abortions and miscarriages considered together) and a higher BC risk in the entire sample (≥1 versus 0: HR = 1.28, 95% CI = 0.98 to 1.67), with a maximum risk among women who had at least three incomplete pregnancies (≥3 versus 0: HR = 2.39, 95% CI = 1.28 to 4.45). HR point estimates seem similar whatever the type of incomplete pregnancy (induced abortions or miscarriages) but were not significant. However, as the number of incomplete pregnancies increased, there was a statistically significant increase in the risk of BC for induced abortions (*P *trend = 0.02), but not for miscarriages. The maximum risk was observed among women who had at least three induced terminations (≥3 versus 0: HR = 3.84, 95% CI = 1.52 to 9.66). Among women who had induced terminations, an age of 20 years or older at first incomplete pregnancy led to a lower risk of BC than an age younger than 20 years (after age 20 versus before HR = 0.50, 95% CI = 0.28 to 0.90). When we considered this risk with respect to the first FTP, the association previously found persisted, but only before the first FTP (HR = 1.77, 95% CI = 1.19 to 2.63). Interestingly, point estimates associated with having miscarriages in the first three months of pregnancy were similar to those associated with induced termination (HR = 1.35, 95% CI = 0.95 to 1.93 and HR = 1.30, 95% CI = 0.93 to 1.82, respectively). There were no differences when stratified by gene.

**Table 3:** Risk of breast cancer associated with incomplete pregnancies and the TMAP score.

Reproductive factors		One woman per family cohort: 39,666 person-years of follow-up					*BRCA1 *mutation carriers: 25,045 person-years of follow-up					*BRCA2 *mutation carriers: 14,621 person-years of follow-up				
		
		Person-years^a^	No. of cases^a^	HR	95% CI	*P *value	Person-years^a^	No. of cases^a^	HR	95% CI	*P *value	Person-years^a^	No. of cases^a^	HR	95% CI	*P *value
Incomplete pregnancies ^b^																
	Never	33,455	219	1.00			21,291	135	1.00			12,164	84	1.00		
	Ever	5,995	155	1.28	0.98-1.67	0.07	3,673	103	1.30	0.95-1.76		2,322	52	1.07	0.64-1.78	
No. of incomplete pregnancies ^b^																
	0	33,455	219	1.00			21,291	135	1.00			12,164	84	1.00		
	1	4,208	103	1.25	0.93-1.69		2,544	70	1.27	0.89-1.82		1,664	33	0.96	0.53-1.73	
	2	1,320	32	1.07	0.69-1.66		841	21	1.08	0.65-1.79		479	11	1.01	0.46-2.24	
	≥3	467	20	2.39	1.28-4.45	0.01	288	12	2.45	1.19-5.04	0.02	179	8	2.69	0.71-10.3	
	Trend			1.19	1.02-1.39	0.03			1.20	1.00-1.43	0.05			1.15	0.84-1.58	
Type of incomplete pregnancies ^b^																
	No incomplete pregnancies	33,384	219	1.00			21,220	135	1.00			12,164	84	1.00		
	Induced abortion only	2,909	71	1.29	0.93-1.81		1,859	50	1.35	0.92-1.99		1,050	21	1.02	0.53-1.96	
	Miscarriage only	2,494	65	1.19	0.83-1.72		1,407	41	1.14	0.73-1.78		1,087	24	1.05	0.54-2.04	
	Induced abortion and miscarriage	535	18	1.49	0.84-2.65		350	11	1.51	0.79-2.91		185	7	1.43	0.45-4.51	
No. of induced abortions^b^																
	0	35,968	286	1.00			22,666	177	1.00			13,302	109	1.00		
	1	2,833	66	1.15	0.83-1.60		1,805	47	1.22	0.84-1.78		1,028	19	0.87	0.45-1.68	
	2	497	16	1.44	0.74-2.78		322	10	1.59	0.75-3.40		175	6	1.47	0.44-4.88	
	≥3	114	7	3.84	1.52-9.66	<10^-3^	82	4	3.31	1.13-9.71	0.03	32	3	7.85	1.74-35.5	0.01
	Trend			1.28	1.04-1.58	0.02			1.32	1.04-1.67	0.02			1.26	0.81-1.95	
No. of miscarriages^b^																
	0	36,419	293	1.00			23,121	186	1.00			13,298	107	1.00		
	1	2,273	64	1.21	0.85-1.72		1,321	41	1.20	0.79-1.82		952	23	1.01	0.50-2.02	
	2	508	12	0.98	0.48-1.99		301	6	0.69	0.27-1.81		207	6	1.54	0.64-3.67	
	≥3	248	7	1.18	0.42-3.30		135	5	1.40	0.44-4.53		113	2	0.86	0.10-7.56	
	Trend			1.07	0.87-1.32				1.04	0.81-1.35				1.09	0.75-1.58	
Age at first induced abortion^c^																
	<20 years	1,094	32	1.00			662	20	1.00			432	12	1.00		
	≥ 20 years	2,350	57	0.50	0.28-0.90	0.02	1,547	41	0.53	0.27-1.02	0.06	803	16	0.40	0.10-1.53	
	No induced abortion	35,968	286	0.74	0.31-1.78		22,666	177	0.70	0.27-1.84		13,302	109	1.10	0.12-10.5	
Age at first miscarriage^c^																
	<20 years	220	5	1.00			202	5	1.00			18	0			
	≥ 20	2,809	78	1.04	0.25-4.28		1,555	47	0.87	0.20-3.73		1,254	31			
	No miscarriage	36,419	293	0.81	0.17-3.99		23,121	186	0.69	0.12-3.93		13,298	107			
Induced abortion relative to the first full-term pregnancy^b^																
	No induced abortion	35,964	284	1.00			22,663	175	1.00			13,301	109	1.00		
	Before first full-term pregnancy	1,835	49	1.77	1.19-2.63	0.01	1,223	33	1.77	1.13-2.77	0.01	612	16	1.88	0.86-4.12	
	After first full-term pregnancy	1,613	42	0.97	0.65-1.45		989	30	1.14	0.72-1.79		624	12	0.55	0.26-1.18	
Miscarriage relative to the first full-term pregnancy^b^ 1.00																
	No miscarriage	36,414	291				23,118	186	1.00			13,296	105	1.00		
	Before first full-term pregnancy	1,138	29	1.07	0.65-1.77		729	20	1.01	0.55-1.84		409	9	0.89	0.36-2.16	
	After first full-term pregnancy	1,896	56	1.05	0.73-1.51		1,031	32	1.02	0.65-1.60		865	24	1.09	0.57-2.08	
Type and length of incomplete pregnancies^b^																
	No abortion	33,384	219	1.00			21,220	135	1.00			12,164	84	1.00		
	Induced abortion only	2,909	71	1.30	0.93-1.82		1,859	50	1.36	0.92-2.00		1,050	21	1.01	0.52-1.95	
	Miscarriage with length ≤3 months	2,492	74	1.35	0.95-1.93	0.09	1,461	45	1.27	0.84-1.94		1,031	29	1.35	0.69-2.67	
	Miscarriage with length >3 months	436	8	0.93	0.41-2.12		253	7	1.17	0.47-2.95		183	1	0.14	0.02-1.24	0.08
TMAP score (not including never pregnant women)^d^																
	]0-0.35]	7,545	141	1.00			4,564	85	1.00			2,981	56	1.00		
	]0.35-0.40]	3,416	88	1.05	0.75-1.48		2,154	59	0.99	0.66-1.48		1,262	29	1.09	0.60-1.98	
	]0.40-0.45]	1,735	51	1.23	0.81-1.86		1,034	30	1.12	0.67-1.87		701	21	1.33	0.68-2.59	
	]0.45-0.50]	589	17	1.53	0.80-2.93		433	14	1.41	0.68-2.92		156	3	1.70	0.37-7.71	
	]0.5-1.00]	1,186	22	1.97	1.19-3.29	0.01	722	12	1.91	1.07-3.42	0.03	464	10	2.04	0.79-5.24	
	Trend			1.16	1.03-1.30	0.02			1.14	0.99-1.32	0.07			1.17	0.95-1.44	

^a ^Not including missing data. ^b^Adjusted for parity (0, 1, 2, ≥3), menopausal status (yes, no), oral contraceptives (never, ever), and gene mutated (*BRCA1*, *BRCA2*). ^c^Adjusted for parity (0,1 2, ≥3), no. of incomplete pregnancies (0, 1, 2, ≥3), menopausal status (yes, no), oral contraceptives (never, ever), and gene mutated (*BRCA1*, *BRCA2*). ^d^Adjusted for menopausal status (yes, no), oral contraceptives (never, ever), and gene mutated (*BRCA1*, *BRCA2*). HR, hazard ratio; CI, confidence interval.

To take into account simultaneously the contrary effect on BC risk of FTPs and pregnancies interrupted within the first three months, we determined the TMAP score defined as the Time of breast Mitotic Activity during Pregnancies. The TMAP score is the sum of pregnancies with a duration greater than or equal to three months multiplied by three plus the sum of the duration (in months) of each pregnancy with a duration of less than three months divided by the sum of the duration of each pregnancy whatever the outcome of the pregnancy. The TMAP score is a time-dependent variable.

Compared with women with a TMAP score of less than 0.35, an increasing TMAP score was associated with a statistically significant increase in the risk of BC (*P *trend = 0.02) and reached 1.97 (95% CI = 1.19-3.29) when the TMAP score was greater than 0.5.

Estimated risks of BC associated with parity, age at first FTP, and history of breast-feeding according to the mutation location were assessed by regions of *BRCA1 *and *BRCA2 *previously defined as homogeneous for the risk of BC [30]. Among *BRCA2 *mutation carriers, no variation of BC risk was found (data not shown). Estimated risks of BC associated with parity and incomplete pregnancy by homogeneous region in *BRCA1 *are shown in Table 4. Parity seems to be associated with a significantly decreased risk of BC only among women with a mutation in LR1 (≥1 versus 0 FTP: HR = 0.27, 95% CI = 0.13 to 0.55) (*P*_interaction _<10^-3^). Similarly, an increasing number of FTPs was associated with a statistically significant decrease in the risk of BC only in LR1 (HR = 0.20, 95% CI = 0.08 to 0.49 for women with at least three FTPs compared with nulliparous women). This protective effect persists whatever the age (HR = 0.33, 95% CI = 0.16 to 0.68 and HR = 0.21, 95% CI = 0.09 to 0.51 before and after age 40 respectively). The HR associated with breast-feeding did not differ between LR1 (ever versus never: HR = 0.73, 95% CI = 0.32 to 1.66) and outside LR1 (HR = 0.86, 95% CI = 0.58 to 1.27) (data not shown). There was also no significant interaction between incomplete pregnancy or age at first FTP and mutation location (data not shown).

**Table 4:** Variation of BC risk associated with full-term pregnancies and incomplete pregnancies according to location of the truncating mutation in *BRCA1*.

Reproductive factors	Location of truncating mutation in *BRCA1 *mutation carriers									
	
	Outside LR1 (16,690 person-years of follow-up)					In LR1 (5,367 person-years of follow-up)				
	
	Person-years^a^	No. of cases^a^	HR	95% CI	*P *value	Person-years^a^	No. of cases^a^	HR	95% CI	*P *value
Parity^b^										
Nulliparous	10,911	21	1.00			3,378	9	1.00		
Parous	5,779	145	1.42	0.77-2.63		1,989	34	0.27	0.13-0.55	<10^-3^
No. of full-term pregnancies^b^										
0	10,911	21				3,378	9	1.00		
1-2	4,218	108	1.63	0.88-3.05		1,354	23	0.32	0.15-0.68	<10^-3^
≥3	1,546	36	0.96	0.48-1.94		635	11	0.20	0.08-0.49	<10^-3^
Full-term pregnancies by attained age^b^										
Nulliparous	10,911	21	1.00			3,378	9	1.00		
Before age 40	4,325	81	1.36	0.72-2.56		1,501	23	0.33	0.16-0.68	<10^-3^
After age 40	1,439	63	1.49	0.70-3.19		488	11	0.21	0.09-0.51	<10^-3^
Incomplete pregnancies ^c^										
Never	14,247	92	1.00			4,419	23	1.00		
Ever	2,404	73	1.43	0.98-2.07	0.06	948	20	0.95	0.47-1.96	
No. of incomplete pregnancies ^c^										
0	14,247	92	1.00			4,419	23	1.00		
1-2	2,254	66	1.36	0.93-2.00		820	16	0.82	0.39-1.73	
≥3	150	7	2.59	1.24-5.40	0.01	128	4	2.05	0.48-8.76	
Type and length of incomplete pregnancies ^c^										
No incomplete pregnancies	14,204	92	1.00			4,419	23	1.00		
Induced abortion only	1,279	40	1.49	0.95-2.36	0.08	431	5	0.65	0.21-2.00	
Miscarriage with length ≤3 months	924	28	1.33	0.79-2.26		428	13	1.25	0.56-2.81	
Miscarriage with length >3 months	153	4	1.12	0.35-3.59		46	2	1.52	0.18-12.5	

^a^Not including missing data. ^b^Adjusted for menopausal status (yes, no) and oral contraceptives (never, ever). ^c^Adjusted for parity (0, 1, 2, ≥3), menopausal status (yes, no) and oral contraceptives (never, ever). HR, hazard ratio; CI, confidence interval.

## Discussion

Our results confirm the existence of a protective effect of an increasing number of FTPs toward BC among *BRCA1 *and *BRCA2 *mutation carriers. This risk reduction, however, appeared to be significant only for women older than 40 years. Additionally, we found some evidence of an association between pregnancies interrupted within the first three months (induced or spontaneous) and an increased risk of BC. This increased risk appeared to be restricted to incomplete pregnancies occurring before the first FTP. Whatever the outcome of the pregnancy, the results show that a first pregnancy before age 20 was associated with a higher risk of BC than a first pregnancy occurring later. We defined the TMAP score to take into account simultaneously the contrary effect of full-term and interrupted pregnancies. We found a significant positive association between the TMAP score and BC risk. All these results appeared to be similar in *BRCA1 *and *BRCA2*. Nevertheless, our results suggest a variation in BC risk associated with parity according to the location of the mutation in *BRCA1*.

Our study has several limitations. First, our results are based on retrospective information obtained from women who opted for *BRCA1 *and *BRCA2 *mutation screening and genetic testing. One assumption that underlies the method of weighting used in our analyses is that the absolute disease risks are well estimated and ascertainment is not dependent on the covariates of interest [43]. This assumption would be violated if any of the factors related to pregnancies changed the likelihood that women might opt to undergo genetic testing. We are unaware of any study that has assessed whether a woman's uptake of genetic testing differs according to these factors and we cannot assess this potential bias [13].

Second, since our data used prevalent cases with some women being interviewed a long time after their BC diagnosis, we cannot exclude that our findings on parity, breast-feeding and incomplete pregnancies are affected by a potential survival bias. However, we could not detect it in our data by performing extra analyses on subsamples of individuals diagnosed or censured within the five-year period before their interview, with a follow-up being counted only during this five-year period. We did not observe differences in our results using this pseudo-incident cohort.

It is well established that increasing parity and early age at first birth are associated with a lower risk of developing BC in the general population. There is evidence that the protective effect of parity may be restricted to women who are over 40 years old [44-47]. The relationship between pregnancy and risk of BC in *BRCA1 *and *BRCA2 *carriers is less clear in the earliest publications [15,16,20,21,23]. Our results are more in line with more recent studies [13,17,22,48] which found a decreased risk associated with an increasing number of FTPs among *BRCA1 *and *BRCA2 *mutation carriers. In agreement with our findings, three of these studies showed a reduced risk of BC only after age 40 years [13,22,48]. Among the studies which assessed the risk of BC associated with the age at first FTP [13,15,17,19-22,48] results are inconsistent and only two studies found a reduced risk among *BRCA1 *or *BRCA2 *mutation carriers associated with a first FTP after age 20 [13,20]. In contrast with our results, the International BRCA1/2 Carrier Cohort Study (IBCCS) study [13] found a variation in this risk by gene mutated. They found that a first FTP after the age of 30 years was associated with a significant decrease in BC risk in *BRCA1 *and a significant increase in *BRCA2 *mutation carriers. Antoniou *et al. *[48] subsequently carried out a similar analysis on 789 *BRCA1/2 *mutation carriers from the UK and found that in *BRCA2 *mutation carriers the risk is higher for those who have their first FTP later, that is, after age 30. We did not find such a variation although our data overlap for about one quarter of our subjects (319 out of 1,337) with those of the IBCCS study.

A number of studies have examined the risk of BC associated with interrupted pregnancies, but there has been some controversy in the past. A collaborative reanalysis of data from 53 epidemiological studies, including 83,000 women with BC from 16 countries, described inconsistent findings across studies and difficulties in evaluating these associations. It was concluded that BC risk did not appear to be associated with an increased number of either spontaneous or induced abortions [10]. Similar results were obtained subsequently from a prospective study of young women [12]. However, numerous studies have suggested that interrupted pregnancies may moderately increase the risk of BC [5-9,49]. Few studies have examined this association in *BRCA1 *and *BRCA2 *mutation carriers. Two studies concluded that BC risk did not appear to be associated with an increased number of either spontaneous or induced abortions [13,15]. Furthermore, Friedman *et al. *observed that among *BRCA2 *mutation carriers, two or more therapeutic abortions resulted in a 64% decrease in BC risk, but not among *BRCA1 *mutation carriers [14]. In 1995, evidence was found that the relative risk conferred by a family history of BC increased with the number of interrupted pregnancies and that this risk was highest for those who had an interrupted pregnancy before the first FTP [50]. Our findings seem consistent with this study. Although, as in many previous studies (for example [51]), a recall bias where BC cases declared interrupted pregnancies more often than controls, would lead to a BC bias away from the null hypothesis. Indeed, we found an increased BC risk associated with an increasing number of induced abortions. However, this risk appeared to be restricted to pregnancies with induced interruptions before the first FTP. This effect may be because the differentiation of mammary cells which occurs during an FTP [52] prevents the carcinogenic effect of subsequent interrupted pregnancies. In addition, our results indicate that spontaneous abortions occurring in the first three months were associated with an increased risk of BC. The difference in risk according to the pregnancy outcome (interrupted versus full-term) and according to the duration of interrupted pregnancy, whatever the nature of the interruption, and our TMAP scores highlight the importance of the duration of pregnancy as a BC risk factor. This is also illustrated by the findings of Vatten *et al. *[53] who reported that the shorter the length of gestation, the higher the BC risk, in a cohort of about 695,000 women. This score could be useful for the individual estimation of BC risk.

When stratified by homogeneous regions, our results suggest a variation of the BC risk associated with parity according to mutation location in *BRCA1*, but not in *BRCA2*. This is the first time that the effects of pregnancy-related factors according to mutation location have been studied. Although, the significance might occur by chance because of a limited power, parity seems to be associated with a significantly decreased risk of BC among women with a mutation in the LR1 region, but not outside this region. Therefore, pregnancies seem to have the same protective effect in LR1 as in the general population, while outside LR1 parity does not seems to have an effect on BC risk.

Although there is no obvious biological hypothesis to explain this variation, one can expect that BRCA1 acts during pregnancy. Indeed, BRCA1 is also involved in cellular anti-proliferation via inhibition of the transcriptional activity of estrogen receptor α (ERα) [54-56]. Interestingly, this mechanism is postulated to occur through a protein-protein interaction involving domains of *BRCA1 *corresponding to regions outside of LR1: that is, the N-terminus (amino acids 1-300) and the C-terminal region [54]. In addition, Ma *et al. *[57] provide evidence for a difference in some hormone-related risk factor profiles between triple negative (TN) and other BC subtypes, especially, in line with a protective effect of parity in all subtypes except in TN. Thus, it would be of interest to study the relation between mutation location and the tumor subtype to determine whether the TN tumors are more often associated with mutations located outside LR1.

## Conclusions

This study confirms the existence of a protective effect of FTPs toward BC among *BRCA1 *and *BRCA2 *mutation carriers which is restricted to women with mutation in the LR1 region for *BRCA1 *mutation carriers. We also showed the importance of the duration of pregnancies as a BC risk factor.

If our findings are confirmed, taking into account environmental and lifestyle modifiers, mutation position might be important for the clinical management of *BRCA1 *and *BRCA2 *mutation carriers and could also be helpful in understanding how *BRCA1 *and *BRCA2 *genes are involved in BC.

## Abbreviations

95%CI: 95% confidence interval; BC: breast cancer; CGH: comparative genomic hybridization; DGGE: denaturing gradient gel electrophoresis; dHPLC: denaturing high performance liquid chromatography; EMMA: enhanced mismatch mutation analysis; ENIGMA: evidence-based network for the interpretation of germline mutant alleles; FTP: full-term pregnancy; HR: hazard ratio; HR2: high-risk region in *BRCA2*; HRM: high resolution melting; LR1: low-risk region in *BRCA1*; LR2: low-risk region in *BRCA2*; MLPA: multiplex ligation-dependent probe amplification; PTA: protein truncation assay; QMPSF: quantitative multiplex polymerase chain reaction of short fragments; qPCR: quantitative polymerase chain reaction; SSCP: single strand conformation polymorphism; TMAP: mitotic activity during pregnancies; TN: triple negative tumors.

## Author information

GENEPSO Collaborating Centers:

Coordinating Center, Hôpital René Huguenin/Institut Curie, Saint Cloud: Catherine Noguès, Emmanuelle Fourme, Rosette Lidereau; Etienne Rouleau, Sandrine Caputo, Shirley Wakselman

Collaborating Centers: Institut Curie, Paris: Dominique Stoppa-Lyonnet, Marion Gauthier-Villars; Bruno Buecher, Institut Gustave Roussy, Villejuif: Olivier Caron; Hôpital René Huguenin/Institut Curie, Saint Cloud: Catherine Noguès, Liliane Demange; Centre Paul Strauss, Strasbourg: Jean-Pierre Fricker; Centre Léon Bérard, Lyon: Christine Lasset, Valérie Bonadona; Centre François Baclesse, Caen: Pascaline Berthet; Hôpital d'Enfants CHU Dijon - Centre Georges François Leclerc, Dijon: Laurence Faivre; Centre Alexis Vautrin, Vandoeuvre-les-Nancy: Elisabeth Luporsi; Centre Antoine Lacassagne, Nice: Marc Frénay; Institut Claudius Regaud, Toulouse: Laurence Gladieff; Réseau Oncogénétique Poitou Charente, Niort: Paul Gesta; Institut Paoli-Calmettes, Marseille: Hagay Sobol, François Eisinger, Laetitia Huiart; Institut Bergonié, Bordeaux: Michel Longy, Centre Eugène Marquis, Rennes: Catherine Dugast; GH Pitié Salpétrière, Paris: Chrystelle Colas, Florent Soubrier; CHU Arnaud de Villeneuve, Montpellier: Isabelle Coupier, Pascal Pujol; Centres Paul Papin, and Catherine de Sienne, Angers, Nantes: Alain Lortholary; Centre Oscar Lambret, Lille: Philippe Vennin, Claude Adenis; Institut Jean Godinot, Reims: Tan Dat Nguyen; Centre René Gauducheau, Nantes: Capucine Delnatte; Centre Henri Becquerel, Rouen: Annick Rossi, Julie Tinat, Isabelle Tennevet; Hôpital Civil, Strasbourg: Jean-Marc Limacher; Christine Maugard; Hôpital Centre Jean Perrin, Clermont-Ferrand: Yves-Jean Bignon; Polyclinique Courlancy, Reims: Liliane Demange; Clinique Sainte Catherine, Avignon: Hélène Dreyfus; Hôpital Saint-Louis, Paris: Odile Cohen-Haguenauer; CHRU Dupuytren, Limoges: Brigitte Gilbert; Couple-Enfant-CHU de Grenoble: Dominique Leroux; Hôpital de la Timone, Marseille: Hélène Zattara-Cannoni; Inserm U900, Ecole des Mines de Paris, ParisTech, Service de Biostatistiques, Institut Curie, Paris: Nadine Andrieu; Inserm U535, Villejuif: Catherine Bonaïti; Inserm U379, Marseille: Claire Julian-Reynier; Inserm

## Competing interests

The authors declare that they have no competing interests.

## Authors' contributions

NA and CN contributed to the design. CN was responsible for the coordination of the study. RL supervised the mutation codification. GENEPSO group members made a major contribution to acquisition of data. JL was responsible for data preparation, conducted the statistical analyses andwrote the manuscript. NA supervised the analyses and participated in writing of the manuscript. All authors contributed to the interpretation and discussion of the findings and revised the manuscript critically and have given final approval of the version to be published.

## Supplementary Material

Additional file 1 **Distribution of mutations**. Distribution of mutations found in the population under study by gene and by type: Truncating mutations (non-sense mutations, frameshift, and all other type of mutations leading to a truncated protein) and 'other type' (missense mutations, in-phase skipping, large rearrangements, partial and entire gene deletions). Click here for file
