# Supplementary material for: Variation in breast cancer risk associated with factors related to pregnancies according to truncating mutation location, in the French National BRCA1 and BRCA2 mutations carrier cohort (GENEPSO)
Source: Breast Cancer Res. 2012 Jul 3;14(4):R99. doi: 10.1186/bcr3218 (PMC3680948; doi:10.1186/bcr3218)
Supplement: Additional file 1 — Distribution of mutations. Distribution of mutations found in the population under study by gene and by type: Truncating mutations (non-sense mutations, frameshift, and all other type of mutations leading to a truncated protein) and 'other type' (missense mutations, in-phase skipping, large rearrangements, partial and entire gene deletions). [file bcr3218-S1.DOC]

Additional table: distribution of mutations

| BRCA1 | | | |  | BRCA2 | | | |
| --- | --- | --- | --- | --- | --- | --- | --- | --- |
| Truncating mutation | | Mutations "other type" | |  | Truncating mutation | | Mutations "other type" | |
| Nucleotide change | No of families | Nucleotide change | No of families |  | Nucleotide change | No of families | Nucleotide change | No of families |
| c.0_80 | 2 | c.1A>C | 1 |  | c.10G>T | 1 | c.3900_3903delGAC | 1 |
| c.0_5407 | 1 | c.1A>G | 3 |  | c.22_23delAG | 1 | c.7007G>A | 2 |
| c.19_47del | 1 | c.117T>G | 2 |  | c.37_44del8 | 1 | c.7007G>C | 1 |
| c.34C>T | 2 | c.140G>A | 6 |  | c.145G>T | 3 | c.7795_7797delGAA | 2 |
| c.66dup | 2 | c.140G>T | 2 |  | c.172G>T | 1 | c.8167G>C | 2 |
| c.671_4185 | 2 | c.181T>G | 13 |  | c.244A>T | 2 | c.8396G>C | 1 |
| c.68_69delAG | 27 | c.191G>A | 10 |  | c.2471T>G | 1 | c.8486A>G | 1 |
| c.81_134del | 4 | c.211A>G | 1 |  | c.250C>T | 4 | c.9117G>A | 1 |
| c.81_4986del | 1 | c.4484G>A | 2 |  | c.262_263delCT | 1 | c.IVS11+1delG | 1 |
| c.81_548dup | 5 | c.4484G>C | 2 |  | c.273C>A | 1 | c.IVS12+594T>G | 2 |
| c.124delA | 1 | c.5074G>C | 1 |  | c.289G>T | 3 | c.IVS14-2A>T | 3 |
| c.144delG | 1 | c.5077_5079delGCT | 1 |  | c.438delA | 1 | c.IVS17-7C>G | 1 |
| c.178C>T | 3 | c.5077_5080delGCTCins | 1 |  | c.469_470delAA | 1 | c.IVS2+1G>T | 1 |
| c.211delA | 4 | c.5077_5080delGCTGins | 1 |  | c.658_659delGT | 1 | c.IVS3+3delA | 1 |
| c.2125insA | 3 | c.5078_5080delGCTGins | 1 |  | c.662_663delTT | 3 | c.IVS6-2A>G | 1 |
| c.220C>T | 1 | c.5123C>A | 7 |  | c.673_676delACTA | 1 | c.IVS7+3A>G | 1 |
| c.241C>T | 1 | c.5272_5592del | 1 |  | c.700delT | 3 | c.IVS8+4A>G | 1 |
| c.329dup | 1 | c.IVS11+1G>A | 4 |  | c.702delC | 1 | total deletion | 3 |
| c.427G>T | 1 | c.IVS12+2del2insG | 1 |  | c.755_758delACAG | 3 |  |  |
| c.442_4357 | 8 | c.IVS18+5G>A | 1 |  | c.771_775delTCAAA | 1 |  |  |
| c.442_547 | 1 | c.IVS19+2delT | 1 |  | c.772C>T | 1 |  |  |
| c.470_471delCT | 1 | c.IVS20+1G>A | 1 |  | c.8002A>T | 1 |  |  |
| c.798_799delTT | 9 | c.IVS21+2T>C | 1 |  | c.994dup | 1 |  |  |
| c.815_824dup | 1 | c.IVS21+4A>G | 1 |  | c.1117C>T | 2 |  |  |
| c.835delC | 1 | c.IVS21-36del510 | 3 |  | c.1228delA | 1 |  |  |
| c.924delC | 1 | c.IVS2-1G>C | 1 |  | c.1231del11ins4 | 1 |  |  |
| c.925A>T | 2 | c.IVS22+5G>C | 2 |  | c.1257delT | 3 |  |  |
| c.928C>T | 1 | total deletion | 2 |  | c.1310_1313delAAGA | 7 |  |  |
| c.962G>A | 1 |  |  |  | c.1327G>T | 1 |  |  |
| c.981_982delAT | 3 |  |  |  | c.1389_1390delAG | 1 |  |  |
| c.984_988 | 3 |  |  |  | c.1593dup | 2 |  |  |
| c.1010dup | 4 |  |  |  | c.1597delA | 2 |  |  |
| c.1067delA | 1 |  |  |  | c.1612_1613delAG | 1 |  |  |
| c.1115G>A | 1 |  |  |  | c.1636delT | 3 |  |  |
| c.1121_1123delCACinsT | 1 |  |  |  | c.1773_1776delTTAT | 1 |  |  |
| c.1121delC | 5 |  |  |  | c.1796_1800delCTTAT | 4 |  |  |
| c.1157_1170del14 | 1 |  |  |  | c.1797_1801delTTATA | 1 |  |  |
| c.1266T>G | 1 |  |  |  | c.1806dup | 2 |  |  |
| c.1292T>G | 2 |  |  |  | c.1813delA | 2 |  |  |
| c.1387delA | 2 |  |  |  | c.1813dup | 3 |  |  |
| c.1389dup | 1 |  |  |  | c.1929delG | 8 |  |  |
| c.1480C>T | 4 |  |  |  | c.2092delC | 1 |  |  |
| c.1483_1498del16 | 1 |  |  |  | c.2175dup | 1 |  |  |
| c.1504_1508delTTAAA | 3 |  |  |  | c.2612C>A | 1 |  |  |
| c.1529C>G | 1 |  |  |  | c.2636_2637delCT | 1 |  |  |
| c.1551delT | 1 |  |  |  | c.2653_2556delGACA | 2 |  |  |
| c.1618G>T | 1 |  |  |  | c.2806_2809delAAAC | 1 |  |  |
| c.1621C>T | 1 |  |  |  | c.2808_2811delACAA | 11 |  |  |
| c.1630C>T | 2 |  |  |  | c.2870delA | 1 |  |  |
| c.1687C>T | 3 |  |  |  | c.2979G>A | 1 |  |  |
| c.1694delA | 1 |  |  |  | c.3195delT | 1 |  |  |
| c.1714G>T | 2 |  |  |  | c.3267_3268delGA | 1 |  |  |
| c.1758_1761delTATA | 1 |  |  |  | c.3366_3367delAA | 1 |  |  |
| c.1881_1894delCAGT | 1 |  |  |  | c.3405C>A | 1 |  |  |
| c.1888delA | 1 |  |  |  | c.3450insAG | 1 |  |  |
| c.1892dup | 5 |  |  |  | c.3545_3546delTT | 6 |  |  |
| c.1930delT | 1 |  |  |  | c.3599_3600delGT | 1 |  |  |
| c.1953_1956delGAA | 2 |  |  |  | c.3680_3681delTG | 1 |  |  |
| c.1953delGAAA | 1 |  |  |  | c.3742_3745delAGTG | 1 |  |  |
| c.1961dup | 2 |  |  |  | c.3744_3747delTGAG | 2 |  |  |
| c.1965C>A | 1 |  |  |  | c.3847_3848delGT | 4 |  |  |
| c.2008G>T | 1 |  |  |  | c.3847delG | 1 |  |  |
| c.2019delA | 3 |  |  |  | c.3860_3863delATAA | 1 |  |  |
| c.2043dup | 1 |  |  |  | c.3865_3868delAAAT | 1 |  |  |
| c.2068A>T | 1 |  |  |  | c.3915delT | 1 |  |  |
| c.2138C>G | 1 |  |  |  | c.4095delT | 1 |  |  |
| c.2269delG | 2 |  |  |  | c.4284dup | 1 |  |  |
| c.2309C>A | 1 |  |  |  | c.4638delT | 3 |  |  |
| c.2359dup | 4 |  |  |  | c.4684C>T | 1 |  |  |
| c.2411delA | 1 |  |  |  | c.4889C>A | 2 |  |  |
| c.2501delG | 1 |  |  |  | c.4889C>G | 6 |  |  |
| c.2561_2565delCTCAG | 3 |  |  |  | c.4926_4935del10 | 2 |  |  |
| c.2572C>T | 2 |  |  |  | c.4965C>G | 1 |  |  |
| c.2603C>G | 1 |  |  |  | c.5066insA | 1 |  |  |
| c.2610delCinsTT | 1 |  |  |  | c.5072dup | 1 |  |  |
| c.2612dup | 2 |  |  |  | c.5208delA | 1 |  |  |
| c.2670delG | 2 |  |  |  | c.5216T>A+delT | 1 |  |  |
| c.2676_2679delAAAG | 1 |  |  |  | c.5303_5304delTT | 1 |  |  |
| c.2679_2682delGAAA | 5 |  |  |  | c.5350_5351delAA | 2 |  |  |
| c.2700_2701delTT | 2 |  |  |  | c.5351delA | 3 |  |  |
| c.2709_2710delTG | 1 |  |  |  | c.5353dup | 1 |  |  |
| c.2722G>T | 3 |  |  |  | c.5482_5486delAAATT | 1 |  |  |
| c.2727_2730delTCAA | 1 |  |  |  | c.5576_5579delTTAA | 1 |  |  |
| c.2761C>T | 1 |  |  |  | c.5582dup | 1 |  |  |
| c.2806_2809delGATA | 1 |  |  |  | c.5592_5593delCA | 1 |  |  |
| c.2814delA | 1 |  |  |  | c.5595_5596deldelAT | 1 |  |  |
| c.2846delG | 1 |  |  |  | c.5623delA | 1 |  |  |
| c.2890G>T | 1 |  |  |  | c.5641_5644delAAAT | 2 |  |  |
| c.2911delC | 1 |  |  |  | c.5645C>A | 6 |  |  |
| c.2951_2952delTT | 1 |  |  |  | c.5665delA | 2 |  |  |
| c.3013delG | 3 |  |  |  | c.5682C>G | 2 |  |  |
| c.3016_3019delCATT | 2 |  |  |  | c.5699C>A | 1 |  |  |
| c.3018_3021delTTCA | 3 |  |  |  | c.5720_5723delCTCT | 1 |  |  |
| c.3143delG | 1 |  |  |  | c.5722_5723delCT | 1 |  |  |
| c.3226_3227delAG | 2 |  |  |  | c.5782G>T | 1 |  |  |
| c.3266delT | 1 |  |  |  | c.5835_5843dup | 3 |  |  |
| c.3289delA | 1 |  |  |  | c.5857G>T | 3 |  |  |
| c.3307dup | 2 |  |  |  | c.5904_5907delAGTC | 1 |  |  |
| c.3326_3329delAAAA | 2 |  |  |  | c.5909C>A | 5 |  |  |
| c.3328dup | 1 |  |  |  | c.5944delA | 1 |  |  |
| c.3329delA | 3 |  |  |  | c.5946delT | 11 |  |  |
| c.3331_3334delCAAG | 2 |  |  |  | c.5980C>T | 1 |  |  |
| c.3331delC | 1 |  |  |  | c.5983dup | 1 |  |  |
| c.3352C>T | 1 |  |  |  | c.5984dup | 1 |  |  |
| c.3355_3359delACTGT | 1 |  |  |  | c.6033_6034delTT | 2 |  |  |
| c.3357delT | 1 |  |  |  | c.6085G>T | 1 |  |  |
| c.3381T>G | 1 |  |  |  | c.6209_6212delAAAG | 4 |  |  |
| c.3403C>T | 2 |  |  |  | c.6235delG | 1 |  |  |
| c.3450dup | 1 |  |  |  | c.6270_6271delTA | 1 |  |  |
| c.3476_3479delTAAA | 1 |  |  |  | c.6275_6276delTT | 4 |  |  |
| c.3477_3480delAAAG | 1 |  |  |  | c.6302delA | 1 |  |  |
| c.3481_3491del | 51 |  |  |  | c.6373dup | 1 |  |  |
| c.3481delG | 1 |  |  |  | c.6396dup | 1 |  |  |
| c.3485delA | 1 |  |  |  | c.6401_6404delATAA | 2 |  |  |
| c.3598C>T | 2 |  |  |  | c.6405_6409delCTTAA | 7 |  |  |
| c.3607C>T | 5 |  |  |  | c.6443_6444delCT | 1 |  |  |
| c.3612delA | 1 |  |  |  | c.6468_6469delTC | 1 |  |  |
| c.3627_3631delinsAGAC | 2 |  |  |  | c.6486_6489delACAA | 1 |  |  |
| c.3627dup | 5 |  |  |  | c.6490C>T | 1 |  |  |
| c.3628_3629delAG | 1 |  |  |  | c.6491_6494delAGTT | 1 |  |  |
| c.3648dup | 1 |  |  |  | c.6527delA | 1 |  |  |
| c.3661G>T | 1 |  |  |  | c.6591_6592delTG | 1 |  |  |
| c.3700_3704delGTAAA | 1 |  |  |  | c.6644_6647delACTC | 7 |  |  |
| c.3730_3737del8 | 2 |  |  |  | c.6656C>G | 2 |  |  |
| c.3730ins50 | 1 |  |  |  | c.6684delA | 1 |  |  |
| c.3748G>T | 2 |  |  |  | c.6833_6837delTCTTA | 1 |  |  |
| c.3756_3759delCTGT | 1 |  |  |  | c.6959delT | 1 |  |  |
| c.3756_3759delGTCT | 8 |  |  |  | c.7047delT | 1 |  |  |
| c.3770_3771delAG | 7 |  |  |  | c.7069_7070delCT | 5 |  |  |
| c.3839_3841delCTCinsC | 1 |  |  |  | c.7187dup | 1 |  |  |
| c.3839_3843delCTCAGin | 13 |  |  |  | c.7208_7211delCCAA | 1 |  |  |
| c.3839_3843delCTCGAin | 1 |  |  |  | c.7248delG | 1 |  |  |
| c.3841C>T | 13 |  |  |  | c.7303C>T | 1 |  |  |
| c.3862G>T | 2 |  |  |  | c.7322delG | 1 |  |  |
| c.3904G>T | 1 |  |  |  | c.7379delA | 1 |  |  |
| c.3926delA | 2 |  |  |  | c.7480C>T | 4 |  |  |
| c.3931_3934delAACA | 3 |  |  |  | c.7558C>T | 2 |  |  |
| c.3937C>T | 2 |  |  |  | c.7636_7645del10 | 1 |  |  |
| c.3937G>T | 1 |  |  |  | c.7671delA | 2 |  |  |
| c.3939_3341delTGA | 1 |  |  |  | c.7680delT | 1 |  |  |
| c.3949_3976dup | 1 |  |  |  | c.7680dup | 3 |  |  |
| c.3952_3955delATTG | 1 |  |  |  | c.8029_8030delGA | 1 |  |  |
| c.3968_3971delAAAT | 1 |  |  |  | c.8053delA | 1 |  |  |
| c.4038_4041delAAGA | 1 |  |  |  | c.8072_8073delCT | 1 |  |  |
| c.4065_4068delTCAA | 17 |  |  |  | c.8140C>T | 7 |  |  |
| c.4120_4121delAG | 1 |  |  |  | c.8247_8248delGA | 1 |  |  |
| c.4128delA | 1 |  |  |  | c.8297delC | 1 |  |  |
| c.4183C>T | 7 |  |  |  | c.8364G>A | 3 |  |  |
| c.4186_4357del | 1 |  |  |  | c.8451T>A | 1 |  |  |
| c.4186_4358 | 1 |  |  |  | c.8463dup | 2 |  |  |
| c.4195_4196delAC | 4 |  |  |  | c.8537_8538delAG | 1 |  |  |
| c.4251_4252delGT | 3 |  |  |  | c.8601insT | 1 |  |  |
| c.4258C>T | 1 |  |  |  | c.8848_8851delAAGG | 1 |  |  |
| c.4282ins39 | 1 |  |  |  | c.8904delC | 3 |  |  |
| c.4327C>T | 21 |  |  |  | c.9019insTCTA | 1 |  |  |
| c.4358_4986 | 1 |  |  |  | c.9026_9029delTATCA | 10 |  |  |
| c.4391delC | 5 |  |  |  | c.9026_9030delATCAT | 2 |  |  |
| c.4391delCTAinsTT | 2 |  |  |  | c.9097delA | 1 |  |  |
| c.4391dup | 1 |  |  |  | c.9097dup | 1 |  |  |
| c.4393delA | 1 |  |  |  | c.9196C>T | 1 |  |  |
| c.4485_4675del | 1 |  |  |  | c.9252dup | 1 |  |  |
| c.4485-4986del | 1 |  |  |  | c.9286C>A | 1 |  |  |
| c.4570_4573delTCTC | 1 |  |  |  | c.9294C>A | 4 |  |  |
| c.4575_4585del | 1 |  |  |  | c.9325insATTA | 2 |  |  |
| c.4617_4618delGGinsTT | 1 |  |  |  | c.9382C>T | 4 |  |  |
| c.4689C>A | 1 |  |  |  | c.9403delC | 1 |  |  |
| c.4757dup | 1 |  |  |  | c.9433_9434delGT | 1 |  |  |
| c.4810C>T | 1 |  |  |  | c.9474delA | 1 |  |  |
| c.4947delAGAinsTTTT | 1 |  |  |  | c.9498delT | 1 |  |  |
| c.4964_4982del19 | 1 |  |  |  | c.9883C>T | 1 |  |  |
| c.4998C>A | 1 |  |  |  |  |  |  |  |
| c.5030_5033delCTAA | 11 |  |  |  |  |  |  |  |
| c.5030delC | 1 |  |  |  |  |  |  |  |
| c.5035_5039delCTAAT | 2 |  |  |  |  |  |  |  |
| c.5038_5042dup | 1 |  |  |  |  |  |  |  |
| c.5044_5048delGAAGAin | 1 |  |  |  |  |  |  |  |
| c.5075_5278 | 1 |  |  |  |  |  |  |  |
| c.5080G>T | 2 |  |  |  |  |  |  |  |
| c.5128G>T | 4 |  |  |  |  |  |  |  |
| c.5137delG | 5 |  |  |  |  |  |  |  |
| c.5194_5277del | 1 |  |  |  |  |  |  |  |
| c.5251C>T | 1 |  |  |  |  |  |  |  |
| c.5260G>T | 4 |  |  |  |  |  |  |  |
| c.5265dup | 56 |  |  |  |  |  |  |  |
| c.5266delC | 1 |  |  |  |  |  |  |  |
| c.527_538del12insGG | 1 |  |  |  |  |  |  |  |
| c.5289dup | 1 |  |  |  |  |  |  |  |
| c.5329_5332+6delinsCA | 1 |  |  |  |  |  |  |  |
| c.5409_5410delTG | 1 |  |  |  |  |  |  |  |
| c.5444G>A | 2 |  |  |  |  |  |  |  |
| c.5503C>T | 3 |  |  |  |  |  |  |  |
| c.5536C>T | 1 |  |  |  |  |  |  |  |
| c.5548delC | 1 |  |  |  |  |  |  |  |
| c.IVS3+1G>A | 1 |  |  |  |  |  |  |  |
| c.IVS5+1G>A | 2 |  |  |  |  |  |  |  |
| c.IVS5+3A>G | 10 |  |  |  |  |  |  |  |
| c.IVS5-2A>G | 2 |  |  |  |  |  |  |  |
